# Seroprevalence of antibodies against SARS-CoV-2 among health care workers in a Portuguese hospital

**DOI:** 10.1097/j.pbj.0000000000000239

**Published:** 2023-12-13

**Authors:** Rogério Ruas, Pedro Palma, Fátima Lamas, Anunciação Ruivo, Rui Malheiro, Rita Ferraz

**Affiliations:** a Infectious Diseases Department, Centro Hospitalar Tâmega e Sousa, Penafiel, Portugal; b Immunohemotherapy Department, Centro Hospitalar Tâmega e Sousa, Penafiel, Portugal; c Institut of Public Health of Porto (ISPUP), Porto, Portugal

**Keywords:** SARS-CoV-2, seroprevalence, health care workers

## Abstract

**Background:**

Health care workers (HCW) are presumably exposed to a higher risk of infection by SARS-CoV-2 and could possibly represent a source of transmission to susceptible patients. Thus, characterization of SARS-CoV-2 infection among HCW is necessary to better understand the determinants of viral transmission and properly implement strategies to prevent dissemination and protect HCW and vulnerable patients. The aim of this study was to estimate the seroprevalence of antibodies against SARS-CoV-2 in a Portuguese tertiary hospital, in the period of July 2020 to March 2021, before the generalized use of the SARS-CoV-2 vaccine, characterize its evolution over time, and identify risk factors associated with seroconversion.

**Methods:**

HCW were approached to collect serum samples for qualitative SARS-CoV-2 antibody testing and completion of an online survey capturing demographics, previous symptoms, and details of health care and community exposure. Odds ratio with bivariable and multivariable logistic regression was used to assess characteristics associated with seroprevalence.

**Results:**

One thousand HCW were included for analysis. Two hundred nineteen HCW (22%) were seropositive for immunoglobulin G against SARS-CoV-2, and 166 (17%) were seropositive for immunoglobulin M, most of whom reported a previous diagnosis of SARS-CoV-2 infection. The risk factors associated with seroconversion included a previous COVID-19 diagnosis, contact with patients, occupational contact with colleagues, and outside contact. However, in a multivariate logistic regression analysis, only a previous diagnosis and outside contact were associated with seroconversion. Seropositivity decreased over time, especially 28 weeks after infection.

**Conclusion:**

HCWs have a high seroprevalence for SARS-CoV-2 infection, probably due to a combination of health care and community exposure. Seropositivity decreases over time, but further studies are needed to better understand our adaptive immune response.

## Introduction

COVID-19 was first detected in December 2019,^1^ and after its rapid spread around the globe, it was declared as pandemic on March 2020.^2^ SARS-CoV-2, the virus responsible for COVID-19, can cause a wide variety of clinical syndromes, ranging from asymptomatic disease, to mild symptomatology, to severe pneumonia, acute respiratory distress syndrome, and death.^3^ The ability to cause asymptomatic disease and be transmissible even in the absence of symptoms has been the main obstacle in controlling the dissemination of the virus.^4^ Given the accelerated rise of the number of cases worldwide, health care workers (HCW) were called to deal with a large number of suspected and confirmed cases of SARS-CoV-2 infection, in many cases in health care facilities already overwhelmed by the burden of disease. As such, HCW are presumably exposed to a higher risk of infection by SARS-CoV-2 and could possibly represent a source of transmission to susceptible patients. Thus, quantification and characterization of SARS-CoV-2 infection among HCW in health care facilities is necessary to better understand the determinants of viral transmission and properly implement strategies to prevent dissemination and protect HCW and vulnerable patients.

Serological assays have become crucial in diagnosing recent or past infection and, when applied to large populations, can help us understand the dynamics of viral transmission. However, although scientific progress has been rapidly developed around SARS-CoV-2, the need to better characterize immune response over time remains.

The aim of this study was to estimate the seroprevalence of antibodies against SARS-CoV-2 in a Portuguese tertiary hospital, characterize its evolution over time, and identify risk factors associated with seroconversion, in the period of July 2020 to March 2021, before the generalized use of the SARS-CoV-2 vaccine, characterize its evolution over time, and identify risk factors associated with seroconversion.

## Methods

**Figure 1.**
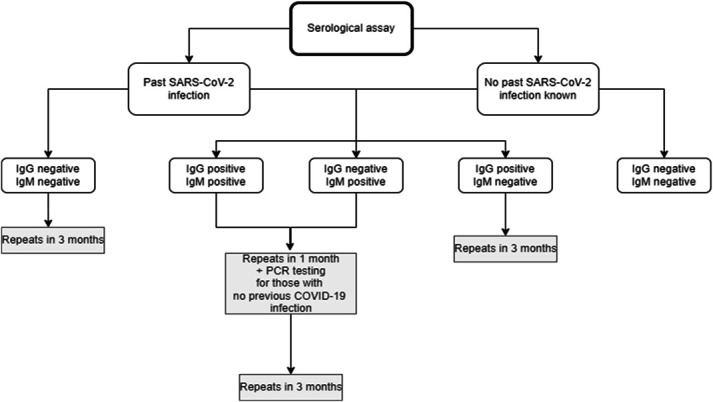
Serological assays according to past SARS-CoV-2 infection

The study population was defined as adults (older than 17 years) registered at the Human Resources department at Centro Hospitalar Tâmega e Sousa (CHTS), a large tertiary hospital in the north region of Portugal with over 4000 beds, servicing a population of over 500.000 people. Exclusion criteria included previous vaccination for COVID-19. From July 2020 to March 2021, all workers were approached by institutional email. After obtaining written informed consent, each participant filled an electronic questionnaire with the following information: demographics, profession, hospital department, previous diagnosis of COVID-19 and symptoms, history of close contact with COVID-19 cases outside the hospital, and history of contact with COVID-19–positive patients. A blood sample was then collected for serological assay. A chemiluminescent microparticle immunoassay (CMIA) was performed on the Alinity i Systems (Abbott, Abbott Park, IL), used for the qualitative detection of immunoglobulin G (IgG) antibodies to SARS-CoV-2 (antinucleocapsid) and immunoglobulin M (IgM) antibodies (antispike protein) in human serum and plasma. According to the results of their serology, participants were then followed as described on Fig. 1.

Baseline characteristics were described through absolute and relative frequency, for categorical variables, and mean and interquartile range for age, the continuous variable. Potential risk factors and seroconversion were analyzed through a multiple logistic regression. Variables significantly different in univariate logistic regression, considering a significance level of 0.05, were included into multivariable logistic regression to identify risk factors of conversion. Sex, personal protective equipment (PPE) formation, occupational contact with a health care worker with COVID-19, contact with a patient with COVID-19, and contact with any individual with COVID-19 outside of the hospital were included in the model. All independent variables were binary. The results were expressed as odds ratio with 95% confidence interval. The Nagelkerke R-squared test and Hosmer and the Lemeshow test were used to evaluate the goodness of fit of the logistic model.

A health care worker was considered to have seroconversion when he or she had at least one reactive assay to either IgM or IgG. Antibody reactivity over time was assessed for workers with previously diagnosed COVID-19 infection, beginning at date of diagnosis, considering both absolute and relative frequency at each 4-week time interval.

Statistical analysis was performed using IBM SPSS Statistics, version 27.

## Results

A total of 1058 HCW enrolled in this study from July 2020 to March 2021, of which 58 were excluded because of previous SARS-CoV-2 vaccination or nonadherence to study protocol. One thousand HCW were included for analysis. At the time of enrollment, 243 (24%) participants reported a previous diagnosis of SARS-CoV-2 infection confirmed by positive PCR test. Of these, 211 (87%) reported symptomatic infection with myalgias being the most common symptom. The baseline characteristics of study participants are described in Table 1.

**Table 1:** Baseline characteristics of study participants

Age (median)	39 (20–66)
Sex (female)	821 (82%)
Professional category	
Physician	157 (16%)
Nurse	381 (38%)
Auxiliary nurse/stretcher-bearer	214 (21%)
Laboratory technician/other technician	94 (9%)
Administrative officers/others	154 (15%)
Previous diagnosis of SARS-CoV-2 infection	243 (24%)
Asymptomatic	32 (13%)
Myalgias	162 (67%)
Anosmia	148 (61%)
Headache	145 (60%)
Dysgeusia	141 (58%)
Cough	122 (50%)
Fever	75 (31%)
Diarrhea	64 (26%)
Odynophagia	64 (26%)
Dyspnea	51 (21%)

Two hundred and nineteen HCW (22%) were seropositive for IgG against SARS-CoV-2, and 166 (17%) were seropositive for IgM, most of whom reported a previous diagnosis of SARS-CoV-2 infection (Table 2). Of note, there were four asymptomatic HCW with no previous history of infection that were seropositive for IgM, who were then tested for SARS-CoV-2 infection and had a positive nasopharyngeal swab for rRT-PCR of SARS-CoV-2. Of those with a previous SARS-CoV-2 infection, 180 (74%) were seropositive for IgG and 137 (56%) were seropositive for IgM.

**Table 2:** Results according to previous SARS-CoV-2 infection

	Total	Previous SARS-CoV-2 infection	No previous infection reported
IgG positive	219 (22%)	180 (74%)	39 (5%)
IgM positive	166 (17%)	137 (56%)	29 (4%)
IgG positive/IgM positive	139 (14%)	118 (49%)	21 (3%)
IgG positive/IgM negative	80 (8%)	62 (26%)	18 (2%)
IgG negative/IgM positive	27 (3%)	19 (8%)	8 (1%)
IgG negative/IgM negative	754 (75%)	44 (18%)	710 (94%)
Total	1000	243 (24%)	757 (76%)

**Figure 2.**
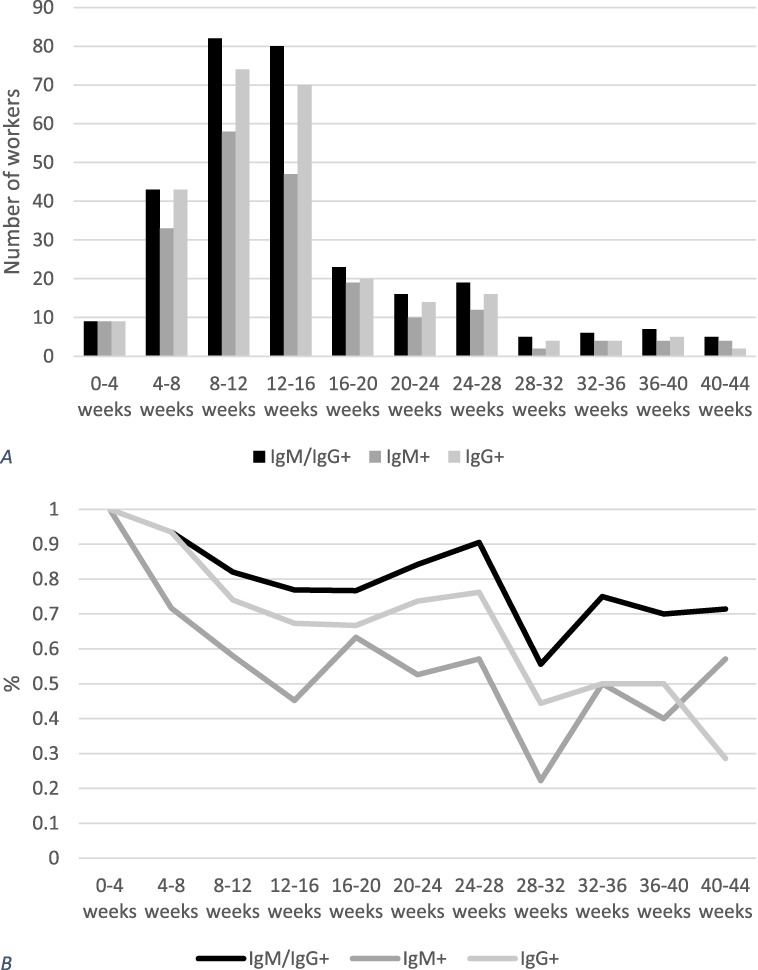
Seroconversion in health care workers with prior diagnosis of COVID-19 over time. (A) Absolute number of health care workers with prior COVID-19 diagnosis with seroconversion. (B) Percentage of health care workers with prior COVID-19 diagnosis tested in each given period with seroconversion; time beginning at date of diagnosis

Antibody reactivity decreased over time as shown on Fig. 2.

The risk factors associated with seroconversion include a previous COVID-19 diagnosis, contact with patients, occupational contact with colleagues, and outside contact. However, in a multivariate logistic regression analysis, only a previous diagnosis and outside contact were associated with seroconversion (Table 3).

**Table 3:** Univariate and multivariate logistic regression analysis of risk factors of seroconversion

Variables	Univariate OR (95% CI)	Multivariate OR (95% CI)
COVID-19 diagnosis	72.30 (46.34–112.78)	66.67 (41.67–100.00)
Contact with patient	2.25 (1.62–3.13)	1.35 (0.80–2.78)
Occupational contact	1.57 (1.18–2.10)	1.31 (0.82–2.09)
Outside contact	2.85 (2.00–4.07)	1.95 (1.12–3.38)
PPE formation	1.14 (0.85–1.52)	
Sex	1.10 (0.76–1.59)	

The Nagelkerke R-squared test was 0.65, and the Hosmer and Lemeshow test *P*-value was .585, meaning that there is no evidence that the model did not fit the data well. The positive predictive value was 0.81, and the negative predictive value was 0.94. Sensitivity was 0.94 and specificity 0.83.

## Discussion

We report the seroprevalence of SARS-CoV-2 in a Portuguese hospital during the first year of the COVID-19 pandemic. The 25% seroprevalence reported is similar to other high-risk cohorts in the United States and the United Kingdom,^5,6^ although a Spanish cohort report much lower seroprevalence (9.3%).^7^ Available data have shown that the seroprevalence of COVID-19 infection is considerably higher among HCWs in comparison with non-HCWs (7.3% vs. 0.4%).^8^

In people with a previous SARS-CoV-2 diagnosis, clinical presentation was very heterogeneous with 13% of asymptomatic cases. IgG serology showed a sensitivity of 74% in this group. Of note, there were four asymptomatic HCW with no previous history of infection that were seropositive for IgM, who then had a positive nasopharyngeal swab for rRT-PCR of SARS-CoV-2. Nurses showed the highest seroprevalence with 38% seropositivity. Interestingly, seropositivity was associated with a previous COVID-19 diagnosis and outside contact, but not with contact within the hospital after multivariate analysis, showing that the driving factor for infection was most likely outside risk and not occupational risk. Infection control measures in place and PPE recommendations thus seem likely to have been effective in protecting HCW from infection.

Of note, seropositivity decreased over time, especially 28 weeks after infection as described in scientific literature.

Our study has several limitations. It is a single-center design with voluntary participation and subject characteristics collected through a self-reported online survey with an irregular adhesion to study protocol. In addition, the study was conducted through a long period and was affected by epidemiological changes in the virus transmission like viral variants and vaccination coverage.

In conclusion, HCWs have a high seroprevalence for SARS-CoV-2 infection, probably due to a combination of health care and community exposure. Seropositivity decreases over time, but further studies are needed to better understand our adaptive immune response. The data presented will be also helpful in better understanding other possible future pandemics associated with airborne or droplet transmission.
